# Localized Characteristics of the First Three Typical Condensation Frosting Stages in the Edge Region of a Horizontal Cold Plate

**DOI:** 10.3390/mi13111906

**Published:** 2022-11-04

**Authors:** Long Zhang, Mengjie Song, Christopher Yu Hang Chao, Chaobin Dang, Jun Shen

**Affiliations:** 1 Department of Energy and Power Engineering, School of Mechanical Engineering, Beijing Institute of Technology, Beijing 100081, China; 2 Department of Building Environment and Energy Engineering & Department of Mechanical Engineering, The Hong Kong Polytechnic University, Hong Kong 999077, China; 3 Graduate School of Engineering, University of Fukui, 3-9-1 Bunkyo, Fukui 910-8507, Japan

**Keywords:** condensation frosting, edge effect, droplet size and distribution, freezing wave propagation, frost layer surface roughness

## Abstract

Condensation frosting usually causes a negative influence on heat exchangers employed in engineering fields. As the relationships among the first three typical condensation frosting stages in the edge regions of cold plates are still unclear, an experimental study on the localized condensation frosting characteristics in the edge region of a cold plate was conducted. The edge effects on the water droplet condensation (WDC), water droplet frozen (WDF) and frost layer growth characteristics were quantitatively investigated. The results showed that the number of droplets coalescing in the edge-affected regions was around 50% greater than in the unaffected regions. At the end of the WDC stages, the area-average equivalent contact diameter and coverage area ratio of water droplets in the edge-affected regions were 2.69 times and 11.6% greater than those in the unaffected regions under natural convection, and the corresponding values were 2.24 times and 9.9% under forced convection. Compared with the unaffected regions, the WDF stage duration in the edge-affected regions decreased by 63.6% and 95.3% under natural and forced convection, respectively. Additionally, plate-type and feather-type frost crystals were, respectively, observed in natural and forced convection. The results of this study can help in the better understanding of the condensation frosting mechanism on a cold plate, which provides guidelines for optimizing the design of heat exchanger structures and system control strategies facing frosting problems.

## 1. Introduction

As a type of efficient heat transfer device, heat exchangers have been widely applied in many engineering applications, such as air source heat pumps, refrigerators, and liquid natural gas air vaporizers [1,2]. However, frost may accumulate on the surfaces of heat exchangers when their surface temperatures are lower than 0 °C and in a humid ambient.

Frost may facilitate heat transfer at the early frosting stage of the heat exchanger due to the increased heat transfer area and turbulent flow caused by frost [3]. 

On the other hand, the gradually accumulated frost may block the air passages of a heat exchanger and increase the thermal resistance between the air and the heat exchanger. As a result, the heat transfer performance of the heat exchanger deteriorates as frosting progresses [4]. Accordingly, the operational efficiency of the related system decreases notably. For example, the heating capacity and coefficient of performance of an air source heat pump unit decreased by 45.1% and 42.5%, respectively, after a 60 min frosting duration [5]. To resolve the frosting problem of heat exchangers, a considerable amount of literature has been published over previous decades.

In general, studies on the frosting characteristics of heat exchangers have mainly focused on the following two aspects. The first is the thermal hydraulic performances of heat exchangers with different fin types under frosting conditions. Liu et al. [6] found that the total heat transfer rate and coefficient of a heat exchanger with perforated fins were 38.9% and 31.8% higher than those with plain fins, but the frost mass of the former was 46.7% higher than that of the latter. Silva et al. [7] demonstrated that frost morphology on the heat exchanger at −5 °C was quite different from that at −10 °C, and the air side pressure dropped significantly depending on frost morphology. Additionally, the air face velocity of the heat exchanger increased as frosting progressed, which may increase the heat transfer coefficient [8]. Moallem et al. [9] developed a semi-empirical correlation of the j factor for a microchannel heat exchanger. The experimental results also indicated that the air side heat transfer coefficient increased at the early frosting stage and decreased at the later frosting stage. The second notable aspect of the studies regarded the frost growth and distribution characteristics on heat exchangers. In most of the related studies, the phenomena of uneven frost distribution along the air flow direction due to the leading-edge effect have been mentioned [10,11,12]. Recently, Zhang et al. [13] suggested that frost on a finned tube heat exchanger could be divided into frost on the edge of windward fins, and the surfaces of fins and tubes; after that, the effects of air temperature, relative humidity, and velocity on these two parts of the frost layer were further quantitatively investigated [14]. A frosting model of a finned tube heat exchanger based on the uneven frost distribution characteristic was also developed [15]. Regarding the complex structures of heat exchangers, it is hard to reveal frosting mechanisms in terms of frosting studies on heat exchangers.

To better understand frosting mechanisms, more attention has been paid to frosting on simple geometries, especially on a flat plate [16]. In terms of the physical process of the early frosting stage, frosting can be divided into desublimation frosting and condensation frosting. Desublimation frosting indicates that water vapor desublimates directly as ice crystals, while condensation frosting indicates that water vapor condenses as water droplets first, and then solidifies [17]. The critical conditions for desublimation and condensation frosting mainly depend on the cold surface contact angle, surface temperature, and water vapor partial pressure [18]. Generally, desublimation frosting usually occurs when the surface temperature [19] or the water vapor partial pressure [20] is extremely low. Since the conditions favorable for condensation frosting are much more accessible than those for desublimation frosting, frosting phenomena in practical engineering are mostly condensation frosting. In terms of the physical process of condensation frosting, it can be divided into four stages. The first is the water droplet condensation (WDC) stage, during which water vapor nucleates as tiny water droplets on the cold surface and keeps growing [21]. The second is the water droplet frozen (WDF) stage, during which water droplets solidify and interdroplet freezing occurs [22]. The third is the frost layer growth (FLG) stage, during which frost crystals grow on the surface of solidified water droplets in three dimensions and form a porous layer [23]. The last is the frost layer full growth (FLFG) stage, during which the frost layer surface melts and freezes periodically, creating a more compact frost layer [24].

In practical engineering, since the defrosting operation usually begins before frosting enters the final stage, the related studies have mainly focused on the first three stages. The WDC stage characteristics of embryo formation [21], droplet growth and coalescence [25], jumping [26], self-propulsion [27], and distribution density [28] have been further investigated. The WDF stage begins with the solidification of one of these water droplets on a cold plate. A complete solidification process of a single water droplet includes subcooling, recalescence, freezing, and subcooling stages [29,30]. Additionally, a notable phenomenon of freezing wave propagation can be observed once one of these droplets solidifies. In 2013, Guadarrama-Cetina first proposed that the freezing wave was mainly propagated by ice bridges, which grew from frozen droplets to liquid droplets [22]. Apart from ice bridges, frost halo [31] and freezing rain [32] have also been demonstrated as other types of propagation mechanisms. The velocity of freezing wave propagation among water droplets was much higher than that of the freezing front of a single water droplet. For the FLG stage, numerical and experimental studies on spatial and temporal characteristics of the frost layer, such as frost thickness, density, and surface roughness, were conducted under different frosting conditions [33,34]. Apart from intelligent models [35], most related frosting models required initial frost thickness and density [36], which are strongly associated with the characteristic parameters at the end of the WDF stage. In particular, FLG characteristics in the edge region are quite different from those in the interior region [37]. However, it can be noted that all the published studies on WDC and WDF stages focused on the interior region of cold plates. As a result, the edge effects on WDC and WDF characteristics are still unclear, as well as the relationships among WDC, WDF, and FLG stages in the edge regions of cold plates.

Therefore, a comprehensive study on the localized condensation frosting characteristics in the edge region of a horizontal cold plate is carried out in this paper. The edge effects on the WDC, WDF and FLG characteristics and their interplay were quantitatively investigated. The specifications of the experimental set-up, experimental procedures, and conditions, as well as data reductions and error analysis, are firstly presented. This is followed by the detailed results and discussion of localized WDC, WDF, and FLG characteristics. Finally, conclusions are given. The results of this paper reveal not only the edge effect on the condensation frosting characteristics, but also the internal relationships among WDC, WDF, and FLG stages in the edge regions of cold plates. Accordingly, the results of this study can help in better understanding of the condensation frosting mechanism on a cold plate, which provides guidelines for optimizing the design of heat exchanger structures and system control strategies facing frosting problems.

## 2. Methodology

### 2.1. Experimental Set-Up

**Figure 1:**
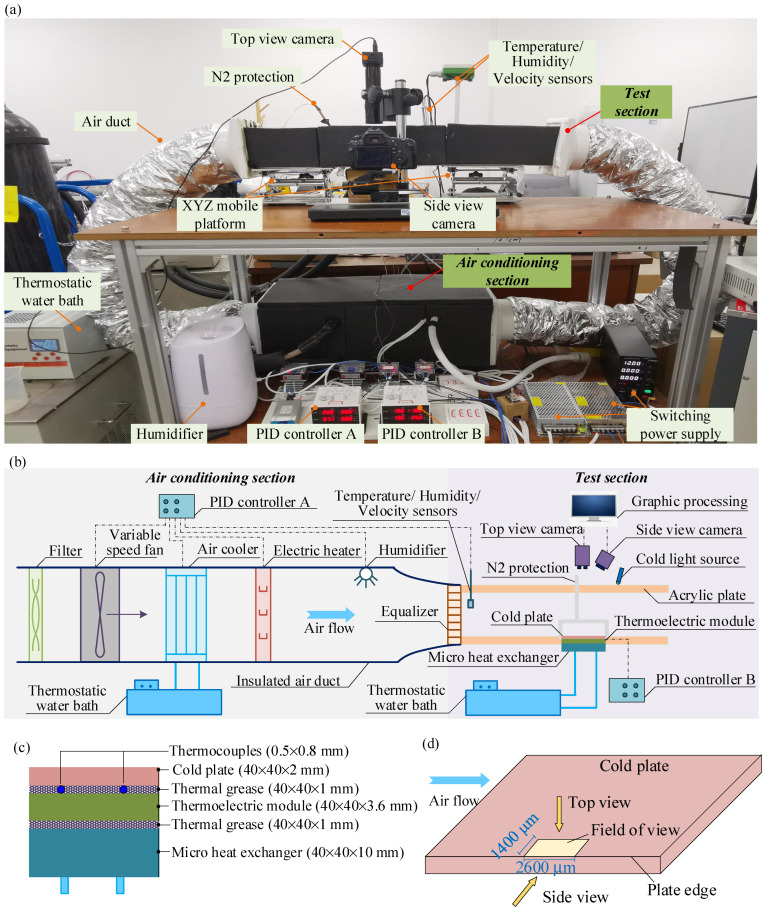
(**a**) photograph of the experimental set-up, (**b**) schematics of the experimental set-up, (**c**) surface temperature measurement, and (**d**) field of view of the experimental cold plate.

A specific experimental set-up was constructed to investigate the localized condensation frosting characteristics in the edge region of a horizontal cold plate. The photograph of the experimental set-up and its schematics are illustrated in Figure 1a,b, respectively. The experimental set-up comprised the air conditioning section and the test section. In the air conditioning section, a constant flow of air with specific temperature, relative humidity, and velocity was processed by a typical air conditioning unit. The air temperature, relative humidity, and velocity were measured by thermocouples (T-TT-36) with an accuracy of ±0.15 °C, humidity sensor (Rotronic HF320) with an accuracy of ±2%, and air velocity sensor (EE650) with an accuracy of ±0.2 m/s, respectively. The air temperature, air relative humidity and air velocity sensors were installed at the inlet of the test section, around 100 mm away from the experimental plate, as illustrated in Figure 1a,b. Using PID controller A, the controlling accuracy of the air temperature, relative humidity, and velocity were ±0.2 °C, ±3.5%, and ±0.2 m/s, respectively. In the test section, the experimental cold plate was horizontally attached to a thermoelectric module and a micro heat exchanger by thermal grease. The dimension of each component is presented in Figure 1c. The static contact angle of the cold plate was 74.6° ± 2.5°. Since the material of the cold plate was copper and its thickness was 2 mm, the difference in temperature between the upper and bottom surfaces of the cold plate was less than 0.1 °C due to little thermal resistance. Therefore, the upper surface temperature of the experimental cold plate could be evaluated by four uniformly distributed superfine thermocouples that were installed at the bottom of the plate. By using PID controller B, the controlling accuracy of the cold plate surface temperature was ±0.2 °C. In general, the air temperature, air relative humidity, air velocity and cold plate temperature could be maintained in the range of 5.0~25.0 °C (±0.2 °C), 30~90% (±3.5%), 0~3 m/s (±0.2 m/s), and −30.0~30.0 °C (±0.2 °C), respectively.

To avoid undesired condensation on the cold plate at the preparation stage of each experiment, an N_2_ protection unit was employed. It was composed of an N_2_ pipe and N_2_ mask, which separated the cold plate from the air at the preparation stages. To better investigate the localized frosting characteristics in the edge region of the cold plate, a top view camera (CCD AO-508U) and a side view camera (Canon-60D) were simultaneously employed. The field of view of the cold plate was around 2600 × 1400 μm, as shown in Figure 1d. The resolutions of the top and side view cameras were 2592 × 1944 and 1920 × 1080, respectively.

### 2.2. Experimental Procedure and Conditions

The complete experiment comprised three stages: the preparation stage, the frosting experiment stage, and the defrosting experiment stage. The detailed experimental procedure in each stage was as follows:

- Preparation: (1) adjust the air temperature, relative humidity, and velocity to the designated values, on the air conditioning unit; (2) place the N_2_ mask on the cold plate to avoid undesired condensation; (3) adjust the cold plate surface temperature to the designated value by the thermoelectric module;
- Frosting experiment: (1) remove the N_2_ mask to allow the processed humid air to directly contact with the cold plate; (2) maintain the air temperature, relative humidity and velocity, and cold plate surface temperature at the designated values;
- Defrosting experiment: (1) turn off the air conditioning unit when the frosting time reached the set value; (2) heat the cold plate by the thermoelectric module to melt frost; (3) wipe away the melted water on the cold plate and clean the cold plate with absolute ethyl alcohol.

To increase the reliability of the experimental results, each experiment was repeated at least three times. In this study, two cases were designed in terms of the typical experimental conditions in related references. Cases 1 and 2 corresponded to natural and forced convection, and the detailed experimental conditions are presented in Table 1.

### 2.3. Data Reductions and Error Analysis

**Figure 2:**
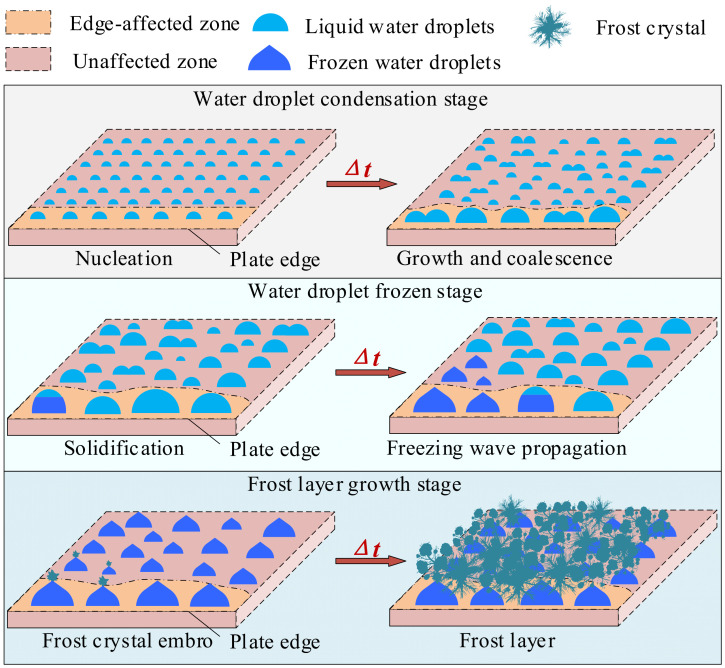
Schematics of the temporal and spatial divisions of condensation frosting stages in the edge region of a cold plate.

In this study, the localized characteristics of the first three stages of a condensation frosting process in the edge region of the experimental cold plate were comprehensively investigated. Based on the visualized data, the schematics of the temporal and spatial divisions of condensation frosting stages in the edge region of a cold plate are illustrated in Figure 2. As seen, the condensation frosting process can be divided into WDC, WDF, and FLG stages, in terms of time sequence. The WDC stage started when humid air contacted the cold plate and a large amount of water vapor nucleated on the cold plate surface. These water droplets gradually grew, and droplet coalescence occurred when two droplets contacted. The WDF stage started when one of the water droplets in the field of view solidified, and interdroplet freezing, also known as freezing wave propagation, could be observed as time passed. The FLG stage started when all the water droplets in the field of view solidified. At that time, frost crystal embryos grew on some early frozen water droplets. As frosting progressed, frost crystals grew in three dimensions and covered each other, creating a porous frost layer. On the other hand, a notable difference in the condensation frosting characteristics was observed in different zones of the cold plate. As a result, the condensation frosting process on the cold plate was further divided into frosting in the edge-affected zone and the unaffected zone regarding its spatial distribution characteristic.

The corresponding characteristic parameters are presented in this section to better understand the temporal and spatial condensation frosting characteristics. Firstly, the contact area of a water droplet can be expressed as:

(1) $$A_{w}=N_{p,\mathrm{in}}\times A_{p}$$

where $N_{p,\mathrm{in}}$ is the number of pixels covered by a droplet, and $A_{p}$ is the area of a pixel. 

The equivalent contact diameter of a water droplet can be expressed as:

(2) $$D_{w}=\sqrt{\frac{4\times A_{w}}{\pi }}$$

The area-average equivalent contact diameter of water droplets can be expressed as:

(3) $${\bar{D}}_{w}=\sqrt{\frac{4\sum_{i=1}^{N_{w}}A_{w,i}}{\pi N_{w}}}$$

where $N_{w}$ is the number of water droplets. 

The coverage area ratio of water droplets to the corresponding region can be expressed as:

(4) $$R_{A}=\frac{\sum_{i=1}^{N_{w}}A_{w,i}}{A_{s}}$$

where $A_{s}$ is the area of the corresponding region. 

During the droplet frozen stage, the average freezing wave propagation velocity can be expressed as:

(5) $$v_{\mathrm{fw}}=\frac{L}{\Delta t}$$

where L is the equivalent distance in the corresponding region, and Δt is the WDF stage duration in the corresponding region. 

Based on the photos captured by the side view cameras, the average water droplet height during WDC and WDF stages can be expressed as:

(6) $${\bar{H}}_{w}=\sqrt{\frac{\sum_{i=1}^{N_{w}}H_{w,i}}{N_{w}}}$$

where $H_{w}$ is the height of a water droplet. 

The average frost layer thickness at the FLG stage can be expressed as:

(7) $${\bar{\delta }}_{f}=\frac{A_{f}}{L^{\prime }}$$

where $A_{f}$ is the area of the frost layer measured by the side view camera, and $L^{\prime }$ is the length of the edge region. 

Based on the average frost layer thickness, the frost layer growth rate can be evaluated by:

(8) $$g_{f}=\frac{{\bar{\delta }}_{f}(t+\Delta t^{\prime })-{\bar{\delta }}_{f}(t)}{\Delta t^{\prime }}$$

where $\Delta t^{\prime }$ is the time interval used for measuring frost thickness. 

The frost layer surface roughness can be evaluated by:

(9) $$RMS_{f}=\sqrt{\frac{\sum_{i=1}^{N_{\mathrm{sp}}}(\delta_{f,i}-{\bar{\delta }}_{f}{)}^{2}}{N_{\mathrm{sp}}}}$$

where $N_{\mathrm{sp}}$ is the number of sampling points, and $\delta_{f,i}$ is the local frost layer thickness of the sampling points. 

The uncertainties of the parameters above can be evaluated using the error propagation formula presented by Rasul et al. [38]. For example, the uncertainty of the frost layer surface roughness can be evaluated by:

(10) $$u_{RMS_{f}}=\frac{\sqrt{\sum_{i=1}^{N_{\mathrm{sp}}}{\left(\frac{\partial RMS_{f}}{\partial (\delta_{f,i}-{\bar{\delta }}_{f})}u_{\left(\delta_{f,i}-{\bar{\delta }}_{f}\right)}\times (\delta_{f,i}-{\bar{\delta }}_{f})\right)}^{2}}}{RMS_{f}}$$

In general, the uncertainties of the characteristic parameters are presented in Table 2.

## 3. Results and Discussion

### 3.1. Localized Water Droplet Condensation Characteristics in Edge-Affected and Unaffected Regions

**Figure 3:**
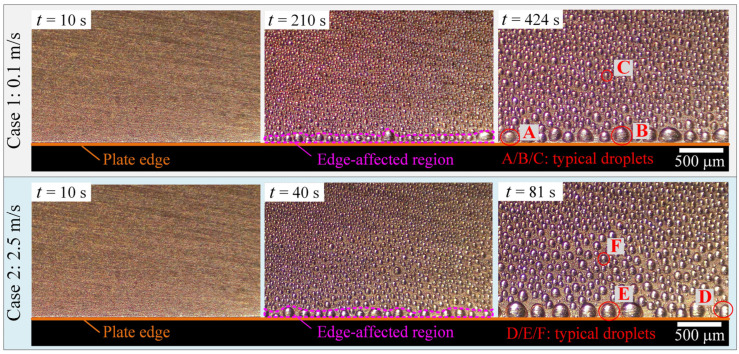
Top-view images of water droplet condensation characteristics.

In this study, the WDC stage started when air with high temperature and humidity contacted the cold plate, and the time point was set as 0 s. Figure 3 compares the top-view images of the localized WDC characteristics in the edge region of the experimental cold plate in Cases 1 and 2. As seen, a large number of nanoscale water droplets nucleated on the cold plate surface at the initial WDC stage and kept growing and coalescing as time passed. A similar phenomenon can also be found in many other related studies [21,25,28]. However, water droplet jumping and self-propulsion were not observed in our study, which was different from [26] and [27], as our experimental cold plate surface was not super-hydrophobic or at a gradient. Additionally, it can be noted that significant differences in water droplet size and distribution characteristics were observed. In general, a single row of water droplets close to the plate edge was much larger and sparser than others, due to the edge effect. Therefore, the region covered by these water droplets was defined as the edge-affected region in this study, as circled by the magenta dash line. The plate edge mainly affected the WDC characteristics through the following three aspects. Firstly, more defects existed in the plate edge, leading to a lower nucleation barrier. Secondly, the plate edge was a joint between the upper and side surfaces of the cold plate, which provided more probability of coalescence of water droplets. Finally, the air side heat and mass transfer rate around the plate edge was much higher due to its geometric singularity. Additionally, it can also be noted that the growth rate of water droplets in Case 2 was much faster than in Case 1 due to the enhanced air side heat and mass transfer rates caused by the air velocity. For example, the water droplets in Case 1 at 210 s were slightly smaller than those in Case 2 at 40 s.

**Figure 4:**
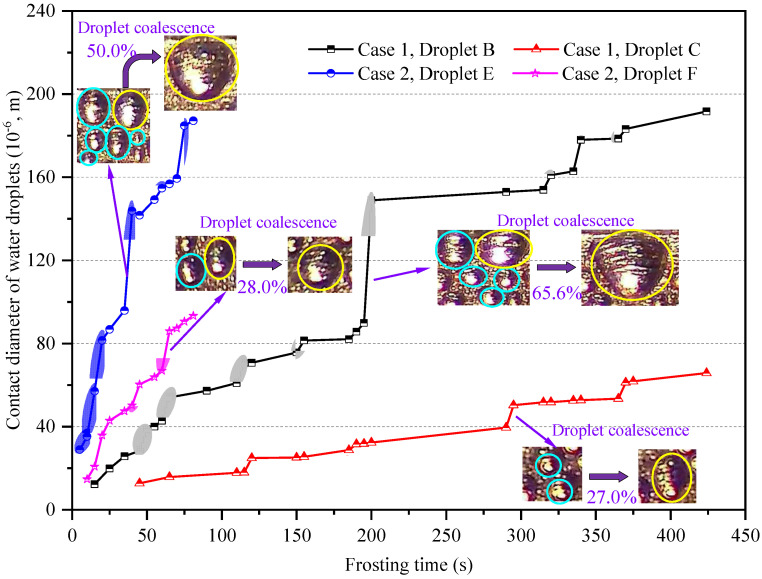
Characteristics of growth and coalescence of water droplets B, C, E, and F.

In particular, the water droplets on the cold plate surface were subcooled at the WDC stage and could solidify at a certain time as they gradually grew up, which was also the indicator of the termination of the WDC stage. Since the plate edge contained more defects, ice nucleation usually occurred first at water droplets close to the plate edge, such as Droplet A in Case 1 at 424 s and Droplet D in Case 2 at 81 s. As a result, the WDC stage duration in Cases 1 and 2 was 424 s and 81 s, respectively. It can be observed that the WDC stage duration was notably shortened by 80.9% when the air velocity increased from 0.1 to 2.5 m/s. To better analyze the droplet dynamics at the WDC stage, four typical water droplets named Droplets B, C, E, and F were selected, as circled by the red line in Figure 3. Droplets B and E represented water droplets in the edge-affected regions in Cases 1 and 2, and Droplets C and F represented those in the unaffected regions in Cases 1 and 2, respectively. The characteristics of growth and coalescence of Droplets B, C, E, and F are further compared in Figure 4.

**Figure 5:**
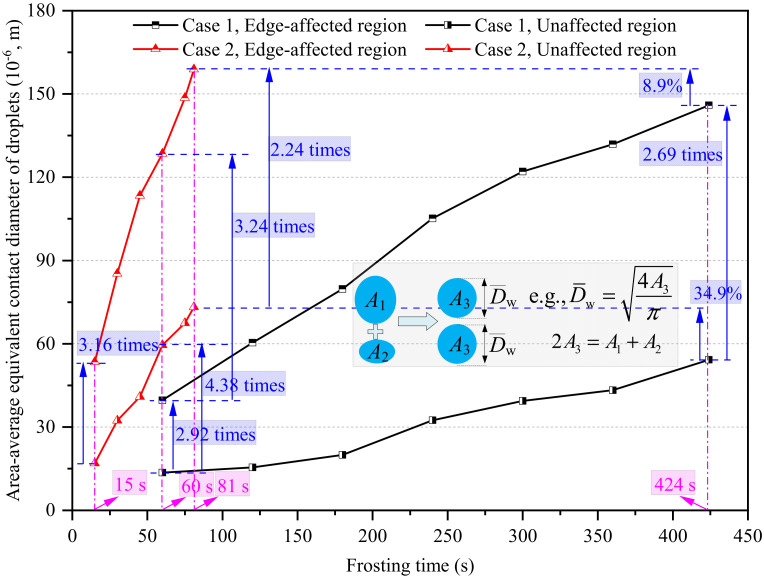
The area-average equivalent contact diameter of water droplets in edge-affected and unaffected regions.

**Figure 6:**
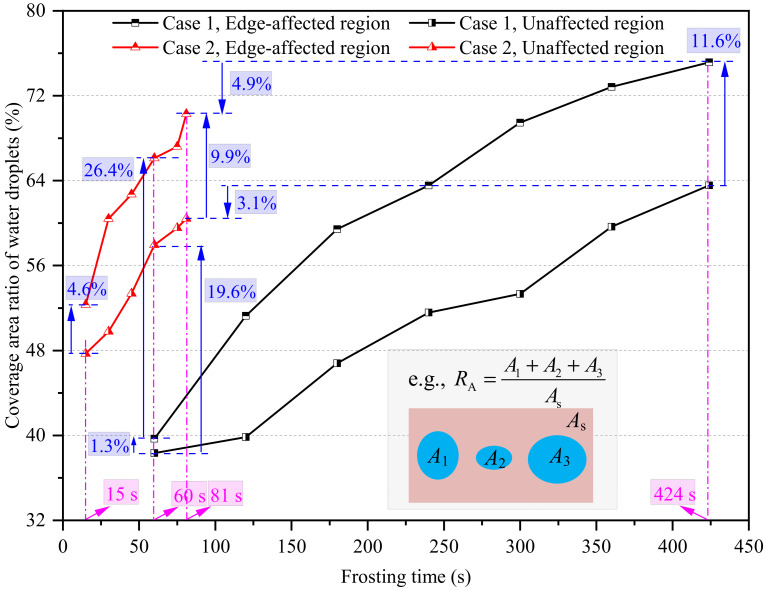
The coverage area ratio of water droplets in edge-affected and unaffected regions.

As shown in Figure 4, the growing processes of Droplets B, C, E, and F were all coupled with droplet coalescence, as indicated by the shadows in the corresponding polygonal lines. For a specific water droplet, its contact diameter notably increased when droplet coalescence occurred. For example, droplet coalescence occurred in the periods of 195 to 200 s, 290 to 295 s, 35 to 40 s, and 60 to 65 s, which increased the contact diameters of Droplets B, C, E, and F, circled by the yellow lines, by 65.6%, 27.0%, 50.0%, and 28.0%, respectively. In addition, the number of droplet coalescences for Droplets B and E were eight and six, which increased by four and three when compared with those for Droplets C and F, respectively. In general, it can be found that the number of droplet coalescences for water droplets in the edge-affected region was around 50% greater than in the unaffected region. Considering the droplet coalescence was a type of rapid droplet growth, the average growth rates of droplet contact diameter for Droplets B and E were 0.45 × 10^−6^ and 2.31 × 10^−6^ m/s, and 31.7% and 100.4% greater than those for Droplets C and F, respectively. To better understand the difference in droplet size and characteristics between edge-affected and unaffected regions, and those between nature and forced convection, the temporal and spatial area-average equivalent contact diameter, and coverage area ratio of water droplets are presented in Figure 5 and Figure 6.

Figure 5 presents the area-average equivalent contact diameter of water droplets in edge-affected and unaffected regions in Cases 1 and 2. As seen, the area-average equivalent contact diameters of water droplets in edge-affected regions were much greater than those in unaffected regions. With respect to Case 1, the area-average equivalent contact diameters of water droplets in edge-affected regions at 60 and 424 s were 39.6 × 10^−6^ and 146.0 × 10^−6^ m, which were 2.92 and 2.69 times greater than those in unaffected regions at the same time points, respectively. With respect to Case 2, the area-average equivalent contact diameters of water droplets in edge-affected regions at 15 and 81 s were 53.4 × 10^−6^ and 164.1 × 10^−6^ m, which were 3.16 and 2.24 times greater than those in unaffected regions at the same time points, respectively. On the other hand, the area-average equivalent contact diameters of water droplets in Case 2 were notably greater than those in Case 1. For example, the area-average equivalent contact diameters of water droplets in edge-affected and unaffected regions in Case 2 at 60 s were 3.24 and 4.38 times greater than those in Case 1 at the same time. However, the WDC stage duration in Case 1 was notably longer than in Case 2. As a result, the area-average equivalent contact diameters of water droplets in edge-affected and unaffected regions in Case 2 were 8.9% and 34.9% greater than those in Case 1 at the end of their respective WDC stages.

Similar to the area-average equivalent contact diameter of water droplets, their coverage area ratio in edge-affected regions was also much greater than that in unaffected regions, as illustrated in Figure 6. At the end of the WDC stages in Cases 1 and 2, the coverage area ratios in edge-affected regions were 2.69 and 2.24 times larger than the corresponding values in unaffected regions, respectively. Additionally, the coverage area ratio of water droplets in Case 2 was notably greater than in Case 1 due to the increased heat and mass transfer rate caused by the increased air velocity. For example, the coverage area ratio of water droplets in edge-affected and unaffected regions in Case 2 at 60 s were 26.4% and 19.6% greater than those in Case 1. However, although the coverage area ratio of water droplets in Case 2 was notably greater than in Case 1 at the same time point, an opposite trend was observed at the end of their respective WDC stages. The coverage area ratios of water droplets in edge-affected and unaffected regions in Case 2 were, in turn, 4.9% and 3.1% smaller than those in Case 1 at the end of their respective WDC stages. The main reason for this was that the WDC stage duration in Case 1 was notably longer than that in Case 2, and the coverage area ratio of water droplets consistently increased as time passed. Therefore, it was found that the final coverage area ratio of water droplets at the end of the WDC stage was not only dependent on the growth rate of water droplets, but also on the WDC stage duration.

**Figure 7:**
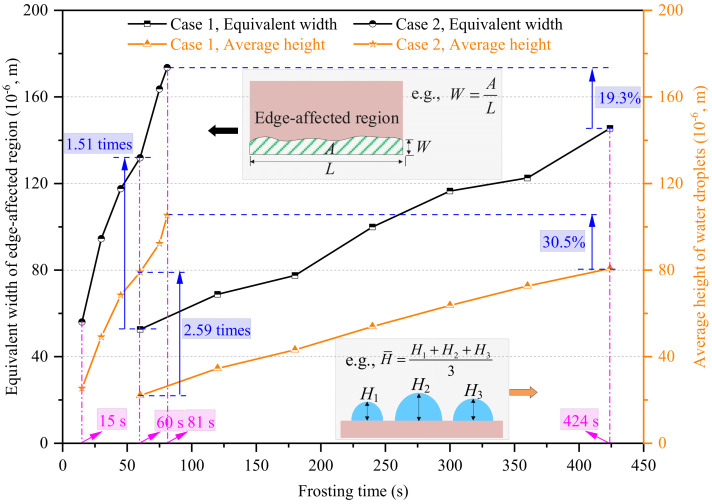
The equivalent width of the edge-affected region and average height of water droplets in the edge-affected region.

Considering the area of the edge-affected region was closely related to the length of the field of view and its shape was not regular, a parameter defined as the equivalent width of the edge-affected region was employed, as presented in Figure 7. The equivalent width of the edge-affected region was used to represent the effective range impacted by the edge effect. As seen, the equivalent width of the edge-affected region consistently increased as time passed and was notably affected by air velocity. At 60 s, the equivalent width of the edge-affected region in Case 2 was 131.9 × 10^−6^ m/s, which was 1.51 times greater than in Case 1. As time passed, the equivalent width of the edge-affected region in Cases 1 and 2 were 145.5 × 10^−6^ and 173.6 × 10^−6^ m/s at 424 and 81 s, respectively. It can be found that the equivalent width of the edge-affected region in Case 2 was only 19.3% greater than in Case 1 at the end of their respective WDC stages because the WDC stage duration in Case 1 was notably longer than in Case 2.

On the other hand, the average height of the water droplets in the edge-affected regions was evaluated by images captured by the side view camera, which was also presented in Figure 7. The variation trend in the average height of the water droplets was similar to the equivalent width of the edge-affected region. In general, the average height of the water droplets in the edge-affected regions consistently increased as time passed. At the end of the WDC stages in Cases 1 and 2, their final values were 80.7 × 10^−6^ and 105.3 × 10^−6^ m, respectively. Although the WDC stage duration in Case 1 was 80.9% longer than in Case 2, the final average height of the water droplets in the edge-affected region in Case 1 was 30.5% smaller than in Case 2, due to the lower heat and mass transfer rate. Similar conclusions can also be found in the study carried out by Zhang et al. [39].

In general, the water droplet size and distribution characteristics at the WDC stage were notably impacted by the plate edge. At the end of their respective WDC stages, the area-average equivalent contact diameter and coverage area ratio of water droplets in edge-affected regions were 2.69 times and 11.6% greater than those in unaffected regions under natural convection, and the corresponding values were 2.24 times and 9.9% under forced convection. Additionally, the differences in the area-average equivalent contact diameter and coverage area ratio of water droplets in edge-affected and unaffected regions, the equivalent width of the edge-affected region, and the average height of water droplets in edge-affected regions, were highly significant between natural and forced convection at the same time point. However, the differences were notably decreased at the end of their respective WDC stages because the WDC stage duration under natural convection was much longer than under forced convection.

### 3.2. Localized Water Droplet Frozen Characteristics in Edge-Affected and Unaffected Regions

**Figure 8:**
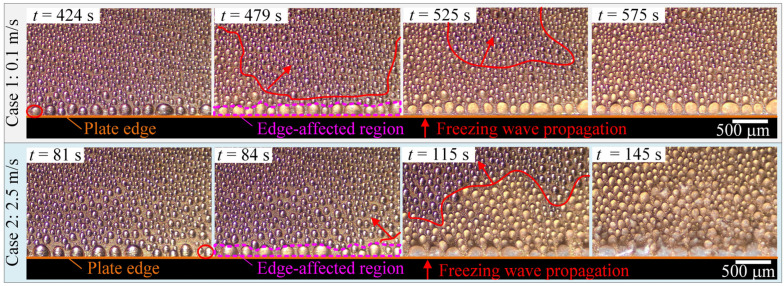
Top-view images of water droplet frozen characteristics.

**Figure 9:**
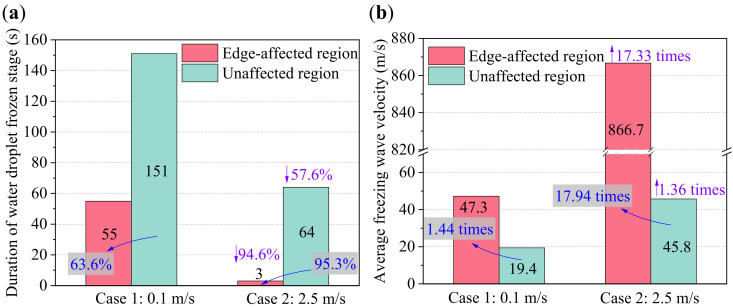
(**a**) Duration of water droplet frozen stage, and (**b**) average freezing wave velocity.

**Figure 10:**
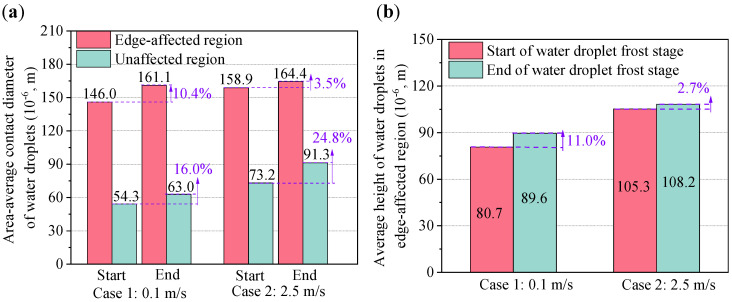
Water droplet size characteristics: (**a**) area-average contact diameter, and (**b**) average height of water droplets.

**Figure 11:**
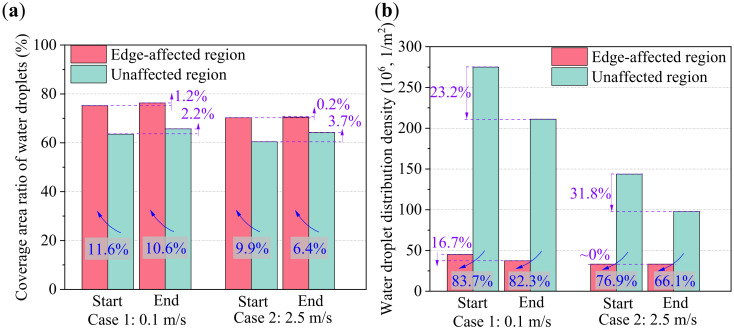
Water droplet distribution characteristics: (**a**) coverage area ratio, and (**b**) distribution density of water droplets.

As mentioned in Section 2.3, the WDF stage started when the ice nucleation occurred for the first time. Figure 8 compares the top-view images of the localized WDF characteristics in the edge region of the experimental cold plate in Cases 1 and 2. It was not difficult to distinguish the solidified water droplets from liquid water droplets due to their different refractive index. As seen, ice nucleation firstly occurred at water droplets in the corner of the edge-affected regions. After that, significant water droplet freezing wave propagation was observed, indicated by the irregular red lines and arrows. It was noted that the water droplet freezing wave propagation velocities in edge-affected regions were much faster than those in unaffected regions. As a result, the WDF stage duration in edge-affected regions was much shorter than in unaffected regions. In general, the difference in freezing wave propagation between edge-affected and unaffected regions was mainly caused by the following three reasons. The first was because the contact diameter and coverage ratio of water droplets in edge-affected regions were greater than those in unaffected regions, which decreased the number of times of freezing wave propagation. The second reason was because there existed a secondary freezing propagation path for the water droplets in the edge-affected region, through the water droplets on the side surface of the cold plate. The third reason was because the heat and mass transfer rates in the edge-affected region were much higher than in the unaffected region, which was also the main reason why the freezing wave propagation in Case 2 was much faster than in Case 1. More quantified data on the WDF stage duration, average freezing wave velocity, water droplet size, and distribution characteristics are presented in Figure 9, Figure 10 and Figure 11.

Figure 9a,b providethe WDF stage duration and average freezing wave velocity in edge-affected and unaffected regions, respectively. With respect to Case 1, the WDF stage duration in edge-affected and unaffected regions was 55 and 151 s, respectively. With respect to Case 2, the WDF stage duration in edge-affected and unaffected regions was 3 and 64 s, respectively. Compared with the unaffected region, the WDF stage duration in edge-affected regions decreased by 63.6% in Case 1, and 95.3% in Case 2, respectively. On the other hand, the average freezing wave propagation velocity in edge-affected and unaffected regions in Case 1 was 47.3 × 10^−6^ and 19.4 × 10^−6^ m/s, and in Case 2 was 866.7 × 10^−6^ and 45.8 × 10^−6^ m/s, respectively. The magnitudes of the average freezing wave propagation velocities in unaffected regions in Cases 1 and 2 were the same as reported in [22], from 10 × 10^−6^ to 100 × 10^−6^ m/s. However, compared with the unaffected region, the average freezing wave propagation velocities in edge-affected regions increased by 1.44 and 17.94 times in Cases 1 and 2, respectively. In general, both the plate edge and air velocity positively impacted the propagation of the freezing wave at the WDF stage. As a result, it was noted that the WDF stage duration in edge-affected and unaffected regions in Case 2 decreased by 94.6% and 57.6%, respectively, when compared with Case 1. In contrast, the average freezing wave propagation velocity in edge-affected and unaffected regions in Case 2 increased by 17.33 and 1.36 times, respectively.

The area-average contact diameter and average height of water droplets at the start and end of the WDF stages in Cases 1 and 2 are illustrated in Figure 10a,b, respectively. As seen, the area-average contact diameters of water droplets in edge-affected and unaffected regions were 161.1 × 10^−6^ and 63.0 × 10^−6^ m at the end of the WDF stage in Case 1, increased by 10.4% and 16.0% when compared with those at the start of the WDF stage. The area-average contact diameters of water droplets in edge-affected and unaffected regions were 164.4 × 10^−6^ and 91.3 × 10^−6^ m at the end of the WDF stage, and increased by 3.5% and 24.8% when compared with those at the start of the WDF stage in Case 2. Additionally, it was also found that the difference in area-average contact diameter of water droplets between Cases 1 and 2 in edge-affected regions was much smaller than that in unaffected regions. The former was around 2.0%, while the latter was 31.0% at the end of the WDF stage. On the other hand, the average heights of water droplets in edge-affected regions at the start and end of the WDF stage in Case 1 were 80.7 × 10^−6^ and 89.6 × 10^−6^ m, and those in Case 2 were 105.3 × 10^−6^ and 108.2 × 10^−6^ m/s, respectively. In other words, the average height of water droplets in edge-affected regions in Cases 1 and 2 at the end of their WDF stages increased by 11.0% and 2.7%, when compared with those at the start of the WDF stages, respectively.

The coverage area ratio and distribution density of water droplets at the start and end of the WDF stages in Cases 1 and 2 are compared in Figure 11a,b. As seen, the coverage area ratios of water droplets in edge-affected and unaffected regions were 76.3% and 65.7% at the end of the WDF stage in Case 1, which increased by 1.2% and 2.2% when compared with those at the start of the WDF stage. For Case 2, the corresponding increase rates in edge-affected and unaffected regions were 0.2% and 3.7%. Additionally, the coverage area ratio of water droplets in edge-affected region was greater than in the unaffected region because the growth rate of water droplets of the former was faster than that of the latter. The coverage area ratio of water droplets in edge-affected regions in Case 1 at the start and end of the WDF stages increased by 11.6% and 10.6%, when compared with those in unaffected regions, respectively. Similarly, the corresponding increase rates were 9.9% and 6.4% in Case 2. The difference in the coverage area ratios of water droplets between edge-affected and unaffected regions decreased as air velocity increased from 0.1 m/s (Case 1) to 2.5 m/s (Case 2).

The distribution density of water droplets was another water droplet distribution characteristic. As seen in Figure 11b, the distribution densities of water droplets in edge-affected and unaffected regions were 37.4 × 10^6^ and 211.1 × 10^6^ 1/m^2^ at the end of the WDF stage in Case 1, and decreased by 16.7% and 23.2% when compared with those at the start of the WDF stage. The corresponding decrease rates were ~0% and 31.8% in Case 2. Additionally, the distribution density of water droplets in edge-affected regions was more than 60% smaller than in unaffected regions due to the faster growth and greater coalescence of water droplets. It was also found that the difference in the distribution density of water droplets between the edge-affected and unaffected regions decreased as air velocity decreased from 0.1 m/s (Case 1) to 2.5 m/s (Case 2).

### 3.3. Localized Frost Layer Growth Characteristics in Edge-Affected Region

**Figure 12:**
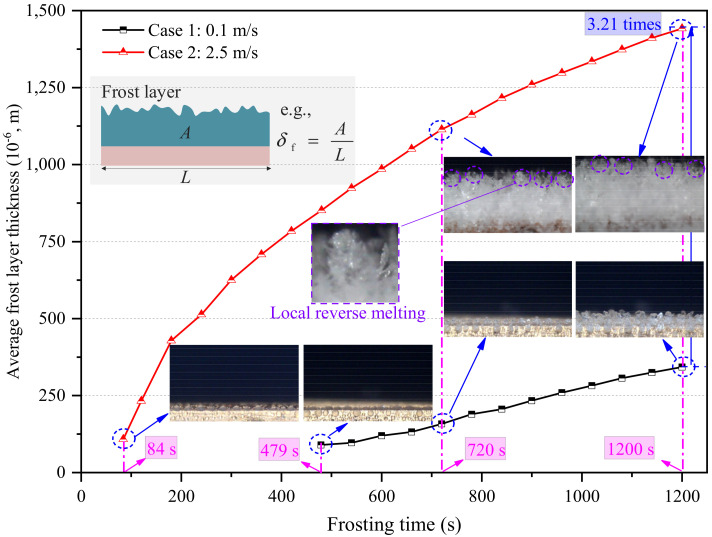
Average frost layer thickness in the edge-affected region.

The FLG stage started when all the water droplets solidified in the field of view. Since the frost layer in the unaffected region was covered by that in the edge-affected region in terms of the side-view images, only the frost layer growth characteristics in the edge-affected regions are presented in this section. Figure 12 presents the average frost layer thickness in the edge-affected regions at the FLG stages in Cases 1 and 2. As seen, the onset time of the FLG stage in Cases 1 and 2 were 479 and 84 s, respectively. Thereafter, frost crystals rapidly grew on the surface of solidified water droplets due to the large temperature difference between air and water droplet surface. As a result, the average frost layer thickness increased as time passed. It can be noted that the average frost layer thickness continued to increase during the remaining frosting time, even though local reverse melting occurred for part of each frost crystal at the later FLG stage. For example, many shining points can be observed in the images of the frost layer at 720 and 1200 s in Case 1. These bright points indicated the local reverse melting of frost crystals. At 1200 s, the average frost layer thickness in edge-affected regions in Cases 1 and 2 was 342.5 × 10^−6^ and 1442.2 × 10^−6^ m, respectively. In other words, the average frost layer thickness increased by 3.21 times after a 1200 s frosting period when the air velocity increased from 0.1 to 2.5 m/s.

**Figure 13:**
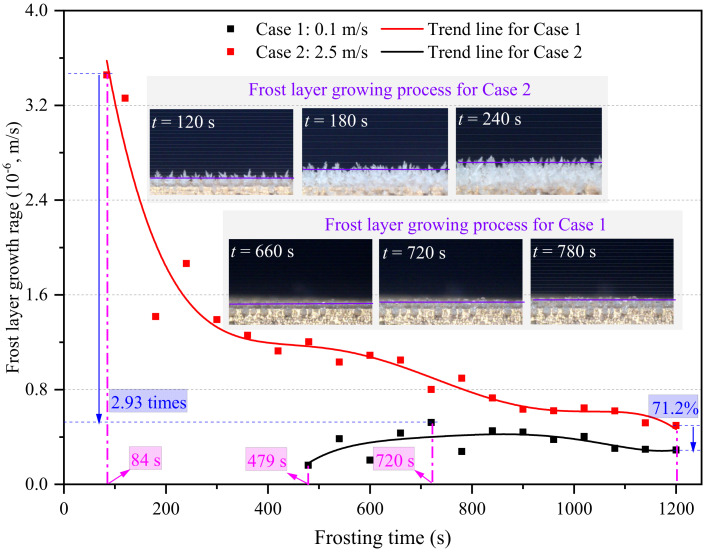
Frost layer growth rate in the edge-affected region.

The frost layer growth rate in the edge-affected regions at the FLG stages in Cases 1 and 2 are compared in Figure 13. As seen, the general variation trend in the frost layer growth rate in Case 1 first increased, and then decreased, as time passed, which was similar to the results reported in [24,34]. At the initial FLG stage in Case 1, the frost layer growth rate was not the maximum, although the frost crystal growth rate later reached the maximum value. The main reason was that many gaps existed among these solidified water droplets, and the frost crystal growth rate in Case 1 was not big enough to balance out these initial gaps. As a result, the average frost layer thickness increased slowly at the initial FLG stage, as demonstrated in Figure 12. The overall frost layer growth rate increased when most initial gaps were filled with frost crystals. At the later FLG stage, the frost layer growth rate decreased as time passed as the surface temperature of the frost layer gradually increased. By contrast, the initial frost crystal growth rate in Case 2 was big enough to balance out the initial gaps among solidified water droplets. Therefore, the frost layer growth rate was maximum at the initial FLG stage and then gradually decreased as time passed. The maximum frost layer growth rate in Case 2 was 3.46 × 10^−6^ m/s at 84 s, which was 2.93 times larger than the maximum frost layer growth rate of 0.52 × 10^−6^ m/s in Case 1 at 720 s. Additionally, the frost layer growth rates in edge-affected regions in Cases 1 and 2 were 0.29 × 10^−6^ and 0.50 × 10^−6^ m/s at the end of the frosting period, respectively. In other words, the frost layer growth rate increased significantly by 71.2% at 1200 s when the air velocity increased from 0.1 to 2.5 m/s.

**Figure 14:**
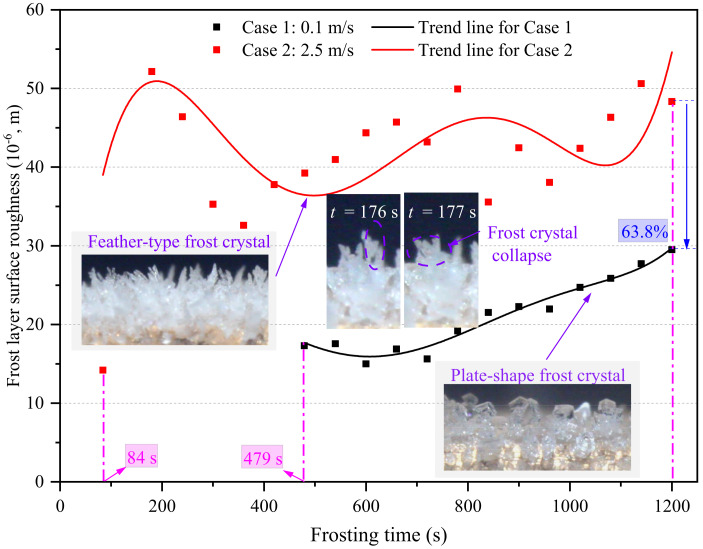
Frost layer surface roughness in the edge-affected region.

Figure 14 shows the frost layer surface roughness in the edge-affected regions at the FLG stages in Cases 1 and 2. As seen, the surface roughness of the frost layer in Case 2 fluctuated more significantly than in Case 1. One reason for this was that the growth rate of frost crystals in Case 2 was much higher than in Case 1, which may have facilitated the uneven growth of each frost crystal. Another reason was that frost crystal collapse occurred more frequently in Case 2 than in Case 1. As illustrated in the images of Figure 14, frost crystal collapse occurring in the period of 176 to 177 s in Case 1 significantly impacted frost layer surface roughness. Additionally, it was also noted that the frost layer surface roughness in Case 2 was much larger than in Case 1. This was mainly because the frost layer thickness in Case 2 was notably larger than in Case 1. At the FLG stage, frost layer surface roughness increased with an increase in frost layer thickness in general. Additionally, the frost crystal in Case 2 was feather-type, which differed from the plate-type frost crystal in Case 1. Feather-type frost crystals also led to a larger frost layer surface roughness when compared with plate-type frost crystals. The frost layer surface roughness in edge-affected regions in Cases 1 and 2 was 29.5 × 10^−6^ and 48.3 × 10^−6^ m/s at the end of the frosting period, respectively. In other words, the frost layer surface roughness increased by 63.8% at 1200 s when the air velocity increased from 0.1 to 2.5 m/s.

## 4. Conclusions

This paper comprehensively studied the localized condensation frosting characteristics in the edge region of a horizontal cold plate. Regarding the characterized condensation frosting stages, the main conclusions can be drawn as follows:

(a) Forced convection may decrease the WDC stage duration and the influence area of the plate edge when compared with natural convection. The WDC stage duration was notably shortened from 424 to 81 s with a decrease of 80.9% when the air velocity increased from 0.1 to 2.5 m/s. The equivalent width of the edge-affected region under forced convection was 19.3% greater than under natural convection, at the end of their respective WDC stages;
(b) The plate edge may facilitate the growth rate of water droplets, which increases their size and coverage area ratio at the early frosting stage. The number of droplet coalescence for water droplets in edge-affected regions was around 50% greater than in unaffected regions. At the end of their respective WDC stages, the area-average equivalent contact diameter and coverage area ratio of water droplets in edge-affected regions were 2.69 times and 11.6% greater than those in unaffected regions under natural convection, and the corresponding values were 2.24 times and 9.9% under forced convection;
(c) Both the air velocity and plate edge positively impacted the propagation of the freezing wave at the WDF stage. Compared with the unaffected region, the WDF stage duration in edge-affected regions decreased by 63.6% under natural convection and 95.3% under forced convection, respectively. The average freezing wave propagation velocities in edge-affected and unaffected regions under natural convection were 47.3 × 10^−6^ and 19.4 ×10^−6^ m/s, and those under forced convection increased by 17.33 and 1.36 times, respectively. Compared with the unaffected region, the average freezing wave propagation velocities in edge-affected regions increased by 1.44 and 17.94 times under natural and forced convection, respectively.
(d) The average frost layer thickness continued to increase at the later FLG stage, even though local reverse melting occurred for part of each frost crystal. The general variation trend in frost layer growth rate under natural convection first increased, and then decreased, while under forced convection it continued to decrease as time passed. The maximum frost layer growth rate under forced convection was 3.46 × 10^−6^ m/s at 84 s, which was 2.93 times larger than the maximum frost layer growth rate of 0.52 × 10^−6^ m/s under natural convection at 720 s. Additionally, the frost layer surface roughness under forced convection fluctuated more significantly than under natural convection, and feather-type and plate-type frost crystals were, respectively, observed in the former and latter.

In general, this paper mainly focused on the localized condensation frosting characteristics in the edge region of a horizontal cold plate under designated experimental conditions, and those for other cold plates with different surface treatments and installation sets and frosting conditions should be further investigated.

## Figures and Tables

**Table 1:** Experimental conditions.

No.	Item	Unit	Case 1	Case 2
1	Air velocity	m/s	0.1	2.5
2	Air temperature	°C	20.0	20.0
3	Air relative humidity	-	50%	50%
4	Cold plate surface temperature	°C	−15.0	−15.0
5	Frosting time	s	1200	1200

**Table 2:** Uncertainties of the characteristic parameters.

Characteristic Parameter	Uncertainty
Equivalent contact diameter of a water droplet	±0.9%
Area-average equivalent contact diameter of water droplets	±2.4%
Coverage area ratio of water droplets	±1.1%
Average height of water droplets	±2.8%
Frost layer thickness	±3.7%
Frost layer growth rate	±2.9%
Frost layer surface roughness	±2.2%

## Data Availability

The data presented in this study are available on request from the corresponding author. The data are not publicly available due to restrictions.

## Nomenclatures

A	area (m^2^)
D	equivalent diameter (m)
$\bar{D}$	area-average equivalent diameter (m)
FLG	frost layer growth
FLFG	frost layer fully growth
g	growth rate (m/s)
H	water droplet height (m)
$\bar{H}$	average water droplet height (m)
L	equivalent distance in the corresponding region (m)
$L^{\prime }$	length of the edged region (m)
N	number
$R_{A}$	coverage area ratio of droplets (%)
RMS	root-mean-square roughness (m)
u	uncertainty (%)
v	velocity (m/s)
*W*	equivalent width of the edge-affected region
WDC	water droplet condensation
WDF	water droplet frozen
Greek symbols	
δ	thickness (m)
$\bar{\delta }$	average thickness (m)
Δt	water droplet frozen stage duration (s)
$\Delta t^{\prime }$	time interval used for measuring frost thickness (s)
Subscripts	
f	frost
fw	freezing wave
p	pixel
p,in	pixels inside a water droplet
sp	sampling points
s	surface of the corresponding region
w	water
